# Simultaneous reconstruction of attenuation and activity for non–TOF PET/MR using MR prior information

**DOI:** 10.1186/2197-7364-2-S1-A30

**Published:** 2015-05-18

**Authors:** Thorsten Heußer, Christopher M Rank, Thomas Beyer, Marc Kachelrieß

**Affiliations:** German Cancer Research Center (DKFZ), Heidelberg, Germany; Center for Medical Physics and Biomedical Engineering, Medical University of Vienna, Vienna, Austria

Accurate quantification of the activity distribution in positron emission tomography (PET) mandates attenuation correction (AC). Unlike in PET/CT, AC in PET/MR is, however, challenging, since information about the attenuation properties of the patient tissue distribution is not available directly. Standard MR-based AC (MRAC) does not account for the presence of bone and, thus, yields an underestimation of the activity distribution. We propose an algorithm to simultaneously reconstruct the activity and attenuation distribution using MR images as anatomical prior information for non time-of-flight PET/MR. The proposed algorithm is an extension of the existing maximum-likelihood reconstruction of attenuation and activity (MLAA). The MR images are used to obtain an initial attenuation map and to derive voxel-dependent expectations on the attenuation coefficients. These expectations are modeled using pre-defined attenuation values and Gaussian-like probability functions. An iterative reconstruction scheme incorporating the prior information on the attenuation coefficients is used to update attenuation and activity distribution in an alternating manner. The algorithm, called MR-MLAA, is evaluated for simulated 2D PET data for two patients with artificial lesions in the head region. The proposed algorithm helps recover bone attenuation information. However, for both patients, some misclassifications of air (considered as bone) and bone (considered as air or soft tissue) were observed. Nevertheless, PET quantification in lesions located close to bone tissue is greatly improved when using MR-MLAA. Errors in activity estimation are reduced to ranges of -9% to +1% whereas MRAC yields errors of -22% to -10%. In conclusion, MR-MLAA has the potential to improve quantification in hybrid PET/MR, especially in regions adjacent to dense bone tissue.

